# Movement toward Environmentally Friendly Pharmaceuticals in Japan

**DOI:** 10.31662/jmaj.2023-0026

**Published:** 2023-09-20

**Authors:** Kei Nagai

**Affiliations:** 1 Department of Nephrology, Division of Clinical Medicine, Faculty of Medicine, University of Tsukuba, Tsukuba, Japan; 2 Department of Nephrology, Hitachi General Hospital, Hitachi, Japan

**Keywords:** greenhouse gas emission, Drug Company, sustainable development goals (SDGs)

## Abstract

As an economic activity, any medical practice involves greenhouse gas (GHG) emissions. In Japan, healthcare accounts for approximately 5% of GHG emissions estimated based on economic costs. In the medical sector, pharmaceuticals account for a large proportion of these emissions. GHG produced during drug manufacturing is generally estimated in three scopes. Scope 1 encompasses direct emissions from equipment and business activities owned by the company; Scope 2 encompasses indirect emissions from the production of energy used in the company’s activities; and Scope 3 encompasses GHG emissions outside the scope of the company’s ownership or control but related to its supply chain (i.e., material procurement, logistics, sales, and disposal). Japanese pharmaceutical companies, similar to companies in other countries, strive to build a sustainable industry. Their efforts have been objectively evaluated, and several companies have been certified by organizations, such as the Carbon Disclosure Project. Regarding biotechnology and the healthcare and pharmaceuticals sectors, 6 of the 14 highest-ranking companies in the world are located in Japan, compared to one or two companies in other nations. Each pharmaceutical company has generally set high emissions goals, although these goals do not necessarily match due to operational differences between companies. Typical strategies to reduce GHG emissions include consolidation of plant facilities, use of renewable energy and eco-cars, simplified packaging of drugs, and shortening of the supply chain. If consumers ignore such companies’ efforts, it could put the brakes on environmental conservation activities in the pharmaceutical sector. Stakeholders, including healthcare providers, could further encourage movement toward environmentally friendly pharmaceuticals by market mechanism through proactively prescribing drugs with less environmental burden. Any clinicians can recognize corporate efforts to protect the environment and contribute to developing environmentally friendly medicine for sustainable growth.

The 2015 Paris Agreement’s central goal to stabilize global mean temperature below 2°C above preindustrial levels will require rapid emission reductions and ultimately net-zero greenhouse gas (GHG) emissions in all sectors of the economy by 2050 ^(1)^. Any medical practice, as an economic activity, involves GHG emissions. In Japan, healthcare accounts for approximately 5% of GHG emissions, estimated from all economic activities ^(2)^. A recent study found that the pharmaceutical industry is more emission-intensive than the automotive industry ^(3)^. Investigations of the breakdown of GHG-induced emissions in healthcare services in Japan revealed that pharmaceuticals have the largest share, at 11.3 mega tonnes of carbon dioxide equivalent (27%) ^(2)^. Despite the heightened urgency of curbing carbon emissions worldwide, the healthcare sector generally―particularly the pharmaceutical sector―has received very little attention from clinical physicians ^(3)^. At a time when climate change is a threat to humans from a public healthcare perspective, reducing the GHG by optimizing the medical disease management strategy and healthcare system is important. Therefore, remaining indifferent to GHG emissions is undesirable for healthcare providers.

Pharmaceutical companies are actively promoting corporate efforts to protect the environment to appeal to their stakeholders and to provide sustainable pharmaceutical products to society. Deciding what environmental measures to take begins with knowing how producing pharmaceuticals impacts the environment. First, I will explain how GHG emissions are calculated. GHGs produced during drug manufacturing are estimated in three scopes based on the proper protocol (Figure 1). Scope 1 encompasses direct GHG emissions from equipment and business activities owned by the company; Scope 2 encompasses indirect GHG emissions from the production of energy used in the company’s activities; and Scope 3 encompasses GHG emissions outside the scope of the company’s ownership or control but related to its supply chain (i.e., fuel combustion and energy use, material procurement, logistics, commuting, sales, and disposal). Japanese pharmaceutical companies, similar to companies in other countries, strive to build a sustainable industry. Particularly, almost all large pharmaceutical companies with sales over several hundred billion yen, including many smaller companies, have calculated Scope 1 + 2 in recent years. However, not all of the companies have calculated up to Scope 3. Among the top 12 Japanese pharmaceutical companies by sales, the mean (interquartile range) proportions of Scopes 1, 2, and 3 are 11.6% (6.2%-16.4%), 10.1% (5.5%-16.1%), and 78.3% (70.6%-86.9%), respectively (Figure 2). Although there are differences among companies, Scope 3 generally accounts for a large percentage of the total. Scope 3 accounts for such a large portion of total GHG emissions because pharmaceutical companies use ingredients purchased from other companies or purchase the drugs themselves for packaging and marketing. Thus, all of their GHG emissions are included in Scope 3. These GHG emissions are often difficult to survey accurately and can thus be described as a “black box” (Figure 1). It should also be cautioned that the figures do not necessarily correspond to those related to pharmaceuticals alone, as many pharmaceutical companies are divisions of larger conglomerate corporations, and estimations may be done on a corporation-wide basis.

**Figure 1.**
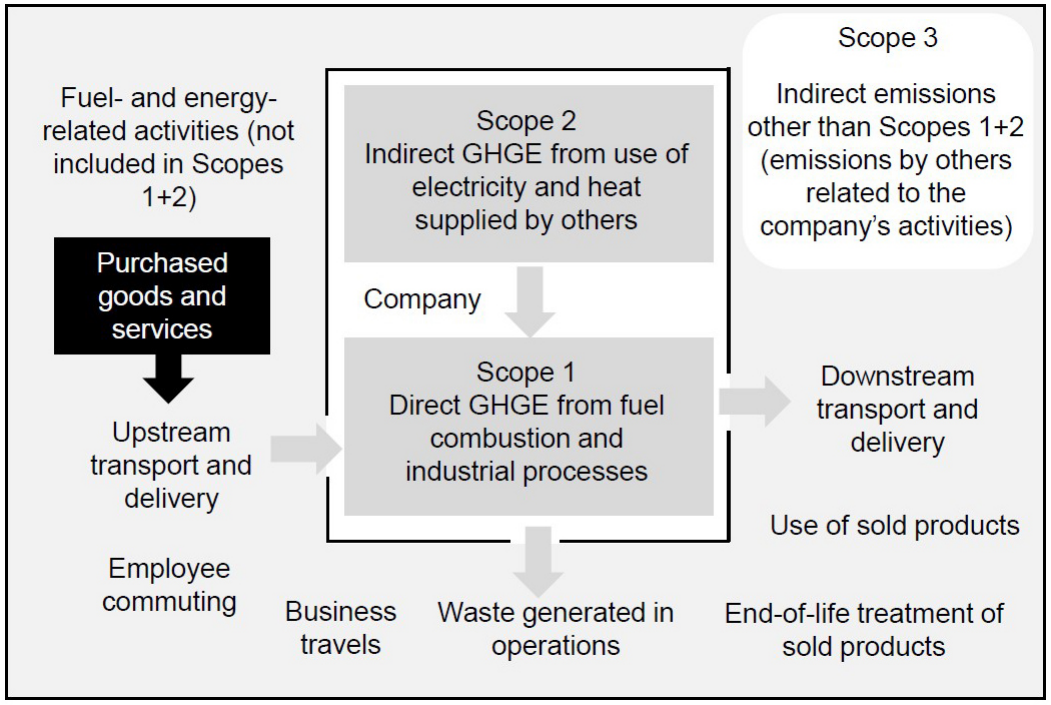
Scopes 1, 2, and 3 for greenhouse gas emission analysis. Regarding environmental assessment, “Scope” refers to the emissions categories specified in the greenhouse gas (GHG) Protocol. Scope 1: Direct GHG emissions by the business itself. Scope 2: Indirect GHG emissions from the use of electricity, heat, and steam supplied by other companies. Scope 3: All indirect emissions other than Scopes 1 and 2. Scope 3 is defined as GHG emissions from all business activities, including suppliers, excluding Scopes 1 and 2. The author prepared this figure based on information published by the Ministry of the Environment, Government of Japan on its website (https://www.env.go.jp/earth/ondanka/supply_chain/gvc/estimate.html).

**Figure 2.**
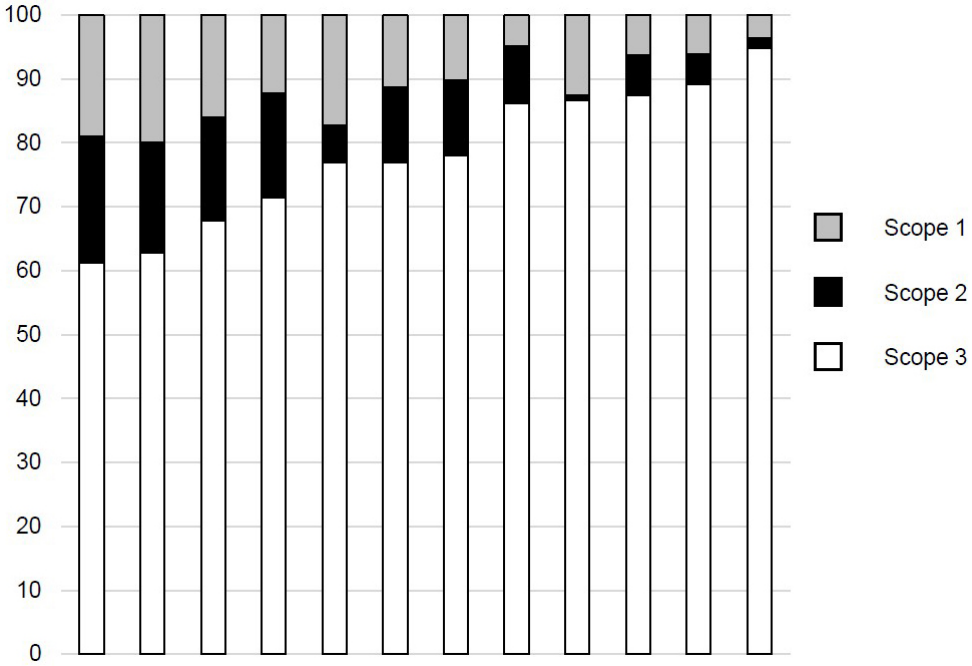
Proportion of Scopes 1, 2, and 3 in large drug companies in Japan. Among the top 12 Japanese pharmaceutical companies (by sales) having annual sales of ≥300 billion yen in 2021, the mean proportions of Scopes 1, 2, and 3 were calculated. The author prepared this figure based on the background information freely available on each company’s website for the public or investors.

While Scope 3 can be difficult to estimate due to the wide range of areas involved, it also represents an extremely promising target for reduction. A company that properly understands Scope 3 can be considered “environmentally conscious.” The efforts of a number of companies have been objectively evaluated, and certification organizations have recognized several companies. The Carbon Disclosure Project (CDP) was established as an international environmental nonprofit organization operating a global environmental disclosure system for corporations and municipalities. The CDP has been managing disclosed data regarding company activities relative to Scope 3 of GHG emissions in the supply chain. In 2022, nearly 20,000 organizations worldwide, including more than 18,700 companies representing half of the world’s market capitalization and more than 1,100 municipalities, disclosed environmental information through the CDP ^(4)^. The CDP asks companies worldwide about their environmental efforts, such as whether they examine the carbon emissions of their activities related to Scope 3 and whether they plan scientific evidence-based measures to reduce emissions. Based on the answers, the CDP ranks the quality of each company’s efforts. In February 2023, the CDP released calculated GHG emissions and specific measures for sustainability and gave progressive companies a rating from A (best) to F (worst). Receiving an A rating requires meeting strict criteria. The initiatives of Japanese pharmaceutical companies are particularly noteworthy. Regarding biotechnology, healthcare, and the pharmaceuticals sector, 6 of the 14 companies worldwide ranking A are located in Japan (of the remaining 8 companies, 2 are in Denmark, and 1 each are located in the United Kingdom, Germany, the United States, the Netherlands, Switzerland, and France).

To ensure the sustainable development of a pharmaceutical company, it is important to promote external environmental activities, build trust with shareholders, raise awareness within the company, and, most importantly, reduce manufacturing environmental costs. Each pharmaceutical company has generally set high emissions goals, although these goals do not necessarily match due to operational differences between companies. The Ministry of Foreign Affairs of Japan aimed to reduce overall GHG emissions to zero by 2050 (i.e., to achieve a carbon-neutral, decarbonized society by 2050). Although GHG emissions are increasing in China and the US, they decreased from 2013 to 2019 in the UK, Australia, and Japan. Regarding the top 12 Japanese pharmaceutical companies presented in Figure 2, although the baseline year for each varies, target emission reductions are set to 20%-55% over the next 25-30 years to achieve carbon neutrality in 2050 (Figure 3). It is also noteworthy that such information is publicly available in an easy-to-understand format for general consumers and investors. Common strategies to reduce GHG emissions include consolidation of product plant facilities, use of renewable energy resources and eco-cars, simplifying drug packaging, and shortening supply chains. In the long view, while energy-efficient plants and power-generation systems should achieve future GHG emission reduction targets, it should be remembered that GHG emissions are required as a fixed capital investment in advance. It would be even better if we could estimate the future economic benefits to a company that results from GHG emission reductions.

**Figure 3.**
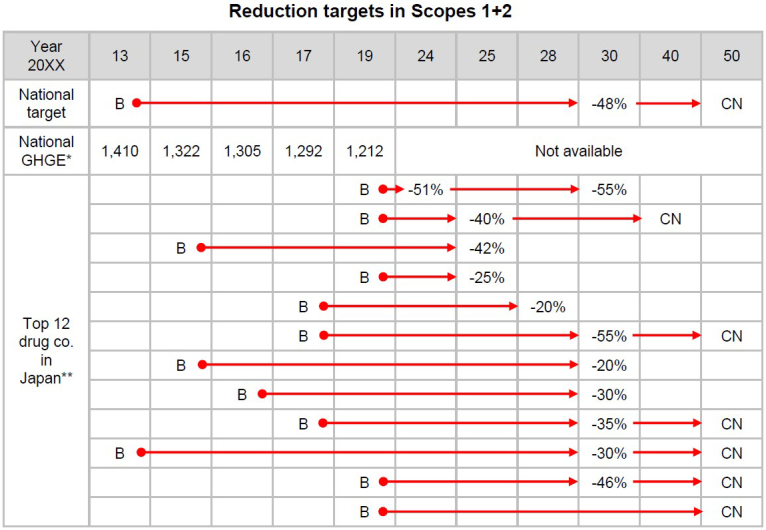
Greenhouse gas emission reduction targets stated by drug companies in Japan. The upper row shows the overall emission reduction targets stated by the Japanese government and actual emissions by 2019 in Japan. Emissions data were taken from the National Institute for Environmental Studies (https://www.nies.go.jp/gio/archive/ghgdata/index.html). The top 12 Japanese pharmaceutical companies stated their greenhouse gas reduction targets in Scopes 1 + 2, and this information is freely accessible on each company’s website for the public or investors. *Greenhouse gas emissions for all of Japan. Units are million ton CO_2_-eq. **Sales > 300 billion yen. Abbreviations: B, baseline year; CN, carbon neutral; GHGE, greenhouse gas emission.

We should learn from the efforts of these Japanese pharmaceutical companies, as Japanese medical professionals. Contrarily, stakeholders, including healthcare providers, could further encourage movement toward environmentally friendly pharmaceuticals by market mechanism through proactively prescribing drugs with less environmental burden. Any clinicians can recognize corporate efforts to protect the environment and contribute to developing environmentally friendly medicine for sustainable growth.

## Article Information

### Conflicts of Interest

None

### Sources of Funding

This work was supported by JSPS grant no. 23K11528 and the Program to Apply for Weaving Diverse Research Skills into an Orchestrated Action to Design Jubilant 100-year Lifetime Society (Tsukuba University, grant #HYL-001) for the preparation of this article. The corresponding author is employed by the University of Tsukuba and Hitachi Ltd., but these organizations did not have any additional role in data collection and analysis, decision to publish, or manuscript preparation.

### Author Contributions

Conceptualization, Investigation, and Writing-Original Draft Preparation: Kei Nagai

### Approval by Institutional Review Board (IRB)

Not applicable.
